# Effects of Dietary Incorporation of Grape Stalks Untreated and Fungi-Treated in Growing Rabbits: A Preliminary Study

**DOI:** 10.3390/ani12010112

**Published:** 2022-01-04

**Authors:** Valéria Costa-Silva, Victor Pinheiro, Anabela Alves, José António Silva, Guilhermina Marques, Jose Lorenzo, Miguel Rodrigues, Luís Ferreira

**Affiliations:** 1 Animal and Veterinary Research Centre, Department of Animal Science, University of Trás-os-Montes and Alto Douro, 5000 Vila Real, Portugal; vpinheir@utad.pt (V.P.); anabela.gaalves@gmail.com (A.A.); jasilva@utad.pt (J.A.S.); 2 Centre for the Research and Technology of Agro-Environmental and Biological Sciences, University of Trás-os-Montes e Alto Douro, 5000 Vila Real, Portugal; gmarques@utad.pt (G.M.); mrodrigu@utad.pt (M.R.); lmf@utad.pt (L.F.); 3 Centro Tecnolóxico da Carne de Galicia, rúa Galicia n 4, Parque Tecnolóxico de Galicia, San Cibrao das Viñas, 32900 Ourense, Spain; jmlorenzo@ceteca.net

**Keywords:** agricultural by-product, grape stalks, growing rabbits, nutritional value, meat quality white-rot fungi

## Abstract

**Simple Summary:**

The use of winery by-products as an animal feed ingredient in rabbit production can enhance the sustainability of this livestock sector by reducing feeding costs and simultaneously diminishing environmental problems related to the management of those by-products. White-rot fungi have been studied for the delignification of lignocellulosic materials due to their potential to decrease the content of lignin. In fact, white-rot fungi also improve the nutritional value due to the deposition of bioactive compounds, acting as a possible biological treatment to enhance the nutritive value of grape stalks. The objective of this work was to evaluate the incorporation of untreated grape stalks and fungi-treated grape stalks in rabbits’ diets.

**Abstract:**

This study aimed to evaluate the effect of the incorporation of untreated grape stalks (U_GS_) and fungi-treated grape stalks (*Lentinula edodes*, T_GS_) in rabbits’ diets. The control group was fed with a control diet without grape stalks (C), two experimental groups were fed on diets with 5% and 10% incorporation of U_GS_ (5U_GS_ and 10U_GS_), and two with 5% and 10% incorporation of T_GS_ (5T_GS_ and 10T_GS_). Rabbits fed with T_GS_ diets showed higher daily weight gain (*p* = 0.034), feed conversion rate (*p* = 0.002), carcass weight (*p* = 0.038), and reference carcass weight (*p* = 0.03) when compared to the control diet. Moreover, animals fed with T_GS_ diets showed an increase in the caecum (*p* = 0.015) and small intestine (*p* = 0.021) lengths and in the total volatile fatty acid content (*p* = 0.005) compared to animals fed U_GS_ diets. Blood triglyceride levels were lower in animals fed with T_GS_ diets compared to U_GS_ (*p* = 0.005) and C (*p* ≤ 0.001) diets (12% and 19% lower, respectively), and a trend to lower cholesterol levels was observed (*p* = 0.071). Meat from rabbits fed with T_GS_ diets had higher levels of linoleic acid, γ-linolenic, ∑ω-6, ∑PUFA, and ∑PUFA/∑SFA ratio compared to rabbits fed with the C diet. Results indicated that grape stalks (U_GS_ and T_GS_) could be effectively used as an alternative raw material in rabbits’ diets without compromising animal performance.

## 1. Introduction

The utilization of winery wastes in animal feeding may represent a viable strategy for livestock production as it could reduce feed costs and make a vital contribution to the possible shortage of raw materials, thus presenting a solution for the environmental problems created by agricultural industries [1,2]. The International Organisation of Vine and Wine (OIV) estimated a global wine production of 294 mhL in 2018 and 60% of that production is carried out by European Union countries. The generated by-products have been estimated to be around 25−30 kg/hL of produced wine, and on average, 4 kg of grape stalks are generated per each hL of produced wine, representing 10–15% of the total produced wastes [3,4]. Currently, grape stalks have been used as fertilizers, but most of the time they are disposed to landfills which, when not attended correctly, can cause environmental problems [5,6]. The nutritional content of grape stalks may not be appealing, limiting its use as a raw material for animal feeding, as they constitute a lignocellulosic fiber material composed mainly of cellulose (30–38%), hemicelluloses (14–21%), and lignin (17–33%) [4,7,8,9]. The high lignin content can be responsible for the limited cell wall digestibility, caused by the strong linkages between lignin and polysaccharides that challenge the enzymatic hydrolysis of cell walls, with consequent negative effects on animal performance [10]. In recent years, there has been an increased interest in the use of biological treatments for lignocellulosic biomass, such as solid-state fermentation with fungi as an alternative for chemical and physical treatments. White-rot fungi are the most efficient microorganisms performing delignification, due to an enzymatic system with extracellular oxidative enzymes (such as laccase, lignin peroxidases, manganese-dependent peroxidase, versatile peroxidase, and others) that catalyze the initial steps of lignin oxidation and depolymerization [9,11]. The modification of lignin structure (delignification) will increase the access to cellulose and hemicellulose and enhance the digestibility of the lignocellulosic biomass. Many studies have evaluated the effect of biological treatment on lignocellulosic biomass with fungi, mainly in sheep [12,13,14,15,16], cattle [17,18,19,20], and rabbits [21,22,23]. In this context, the objective of this study was to evaluate the dietary incorporation of untreated and fungi-treated grape stalks with Lentinula edodes on the diets of growing rabbits.

## 2. Materials and Methods

The experimental trial was conducted according to the Portuguese legislation (Ports. no. 1005/92, 214/08, 635/09) on animal welfare. The ethical committee of the University of Trás-os-Montes and Alto Douro (ORBEA, Animal Welfare Authority) approved the experimental protocol (Process number: 1058-e-DZ-2019).

### 2.1. Untreated and Treated Grape Stalks

Grape stalks (GS) were collected after the destalking process in the region of Trás-os-Montes, Murça, district of Vila Real, Portugal. Grape stalks were dried at 40 °C in an air forced oven (Venticell, MMM Group, Munich, Germany), then ground by a hammer mill (Retsch SM 100, Haan, Germany) at 1 cm and stored for subsequent processing. The stored material was divided into two parts, one designated untreated grape stalks (U_GS_) and stored until the rabbit feed production, and the other one treated (T_GS_) with the basidiomycete *Lentinula edodes* from the culture collection of the Laboratory of Mycology and Soil Microbiology of the University of Trás-os-Montes and Alto Douro, Vila Real, Portugal. *Lentinula edodes* was selected based on the results obtained in a preliminary study (unpublished data) that showed that this fungal strain had a greater potential for the improvement of the nutritional value of grape stalks for rabbit feeding. For the solid-state fermentation (SSF) process, approximately 1 kg of humidified grape stalks was placed in several breathable autoclaving bags (filter type T; filter pore size of 0.2 µm; Unicornbags, Plano, TX, USA), autoclaved (121 °C for 30 min) in an autoclave machine (MLS-3781L-PE, Panasonic Healthcare, Gunma, Japan), cooled, and inoculated with 40 g of grain spawn of *L. edodes* at 28 °C for 50 days.

### 2.2. Experimental Diets

Diets were formulated according to the recommendations of De Blas and Mateos [24] for growing rabbits. Two diets with 100 and 50 g/kg of incorporation of untreated grape stalks (10U_GS_ and 5U_GS_, respectively), two diets with 100 or 50 g/kg of incorporation of fungi-treated grape stalks (10T_GS_ and 5T_GS_, respectively), and a control diet without incorporation of grape stalks were used. The feed ingredients (g/kg, as fed) and their chemical composition are shown in Table 1. 

### 2.3. Animals and Experimental Design

Fifty hybrid (New Zealand × Californian) male rabbits weaned at 35 days of age with an average body weight of 1091 ± 56.3 g were randomly assigned to five experimental treatment groups (10 rabbits/diet). Animals were kept in a closed air-conditioned building maintained between 18 and 23 °C and received 12 h of light daily. The experimental diets were restricted to 90 g/day on the first week and 100 g/day on the second week to avoid digestive problems, as GS were never used on rabbits feeding, and animals were then provided ad libitum until the end of the experiment. No animals died during the experimental period.

### 2.4. Growth Performance Trial

During the experimental period (from 35 d until 66 d), individual live weight and feed intake per cage were measured and the weight gain, daily feed intake, and feed conversion ratio were calculated. No animals died during the trial. Feed consumption was calculated as grams per rabbit per day. Refusals from each cage were collected daily, weighed, and taken into consideration for the calculation of feed consumption and feed conversion ratio (g feed/g gain).

### 2.5. Digestibility Trial

The coefficients of total tract apparent digestibility (CTTAD) of dry matter (DM), organic matter (OM), neutral detergent fiber ash-free (NDFom), crude protein (CP), and ether extract (EE) were measured in six randomly selected rabbits from each experimental diet were determined from 55 to 59 d of age during the growing trial according to the European standardized method [25]. Feces were collected using nylon net placed under each individual cage to avoid urine contamination. Samples of feeds and feces were collected individually and then stored at −20 °C for subsequent chemical analysis. Feed consumed was registered weekly and total fecal excretion was quantified daily from each cage for further calculations.

### 2.6. Chemical Analysis

All samples (feed and feces) were dried at 60 °C to a constant weight in an air forced oven (Venticell, MMM Group, Munich, Germany). The samples were grounded over a 1 mm screen (Tecator Cyclotec 1093 Sample Mill, Foss SA, Sweden) and prepared for chemical analysis. AOAC [26] procedures were used to determine DM (no. 934.01), OM and ash (no. 942.05), ether extract (EE, no.920.39), total dietary fiber, soluble and insoluble fiber (no. 991.43), and total N as per the Kjeldahl method (954.01). The CP content was calculated as N crude protein (CP, no. 954.01). The NDFom was determined without the use of sodium sulfite and α-amylase according to the methodologies proposed by Robertson and Van Soest [27] and Van Soest et al. [28]. 

### 2.7. Slaughtering, Sampling, and Post-Slaughter Analysis

At 66 days of age, the animals were slaughtered by cervical dislocation. The slaughtering and carcass dissection procedures followed the World Rabbit Science Association recommendations described by Blasco and Ouhayoun [29]. 

#### 2.7.1. Blood Analysis

Blood samples were collected during slaughtering directly from the jugular vein into tubes containing ethylenediaminetetraacetic acid tripotassium (K3EDTA; Sigma Company, St. Louis, MO, USA). Hematologic parameters were measured using a hematology analyzer and the respective reagent kit supplied by the manufacturer (Procyte Dx, IDEXX Laboratories, Westbrook, ME, USA). The hematology analyzer combines three major technologies: laser flow cytometry, optical fluorescence, and laminar flow impedance. The hematologic parameters evaluated were erythrocyte, haemoglobin, haematocrit, lymphocytes, monocytes, eosinophils, basophils, and reticulocyte count. For serum biochemistry, samples were let to coagulate and centrifuged at 3500 rpm for 15 min and serum was separated and stored at −20 °C till analyzed. Serum biochemical parameters were measured using automated biochemistry analyser using reagents, calibrators and controls supplied by the manufacturer (Daytona, Randox Laboratories Ltd., CrumLin, UK). The following serum biochemical parameters were estimated: triglycerides, cholesterol, urea, creatinine, aspartate aminotransferase, alanine, albumin, and total protein.

#### 2.7.2. Caecal and Gastrointestinal Parameters

Immediately after slaughter, pH of caecal and stomach content, full and empty gastrointestinal tract weight and length, scapular, pelvic and perivisceral fat, liver and kidneys weights were registered. Samples from the small intestine (duodenum, jejunum, and ileum), liver, and kidney were collected and fixed by immersion in 10% neutral formalin for further analysis (villus and viscera observation). For volatile fatty acids (VFA) analysis, samples of caecal content were collected and frozen. Samples of caecal contents were centrifuged at 10,000 rpm for 15 min at 4 °C and 5 mL of supernatant were collected and was added 0.5 mL of pivalic acid. Then, 4 mL of this solution was mixed with 1mL of H_3_PO_4_ 25% and were centrifuged at 10,000 rpm for 10 min at 4 °C. The supernatant was collected for volatile fatty acid (VFA) analysis. VFA concentrations were determined by gas-liquid chromatography (Shimadzu GC-141 B, Kyoto, Japan) using pivalic acid as an internal standard according to the procedures of Czerkawski [30]. Separation of acetate, propionate, butyrate, and valeric was accomplished on a capillary column (Supelco Nukol, 30 m × 0.25 mm, df 0.25 µm), operated at 135 °C using helium as the carrier gas. The injection temperature was 210 °C. Quantification of the acids was performed using a flame ionization detector at 180 °C connected to an integrator (Shimadzu C-R6A, Kyoto, Japan). 

#### 2.7.3. Villus and Viscera Observation

Samples collected and fixed by immersion in 10% neutral formalin during slaughtering were processed in an automatic tissue processor (Hipercenter XP, Shandon, Cornelius, NC, USA). Samples were dehydrated in increasing ethanol concentrations, cleared in xylene, embedded in paraffin wax (Histoplast, Shandon, Cornelius, NC, USA), and sectioned to a thickness of 3 μm. The sections were routinely stained with hematoxylin and eosin [31]. Slides were evaluated on a stationary digital camera (DXM1200, Nikon, Tokyo, Japan) using optical lens no 4 to measure the height, tip width, crypt depth, and muscle layer thickness. Fifteen villi per animal were assessed and the reported mean values were based on these measurements. A digital program (Digimizer, MedCalc Software Ltd., Ostend, Belgium) was used to measure the characteristics of crypts. 

#### 2.7.4. Carcass Parameters

Hot carcass weight (HCW) did not include blood, skin, distal parts of the tail, fore and hind legs, gastrointestinal and urogenital tracts. After being chilled at 4 °C for 24 h, carcass weight (CCW) was recorded, and pH (pH24) and color carcass were assessed in the left biceps femoris muscle (BFM). The pH was measured directly in the muscle with a 5-mm glass penetration pH electrode (pH Meter 632, Metrohm, Herisau, Switzerland) and the carcass colors were measured in accordance with Ouhayoun and Dalle Zotte [32] using a color measurement instrument (CR-300 Chroma Meter, Minolta, Osaka, Japan) in the CIELAB color space: lightness (L*), redness (a*), and yellowness (b*). The weight of the hindleg, each thigh, loin, rib, paw, head, liver, kidneys, organs of thorax and neck (LHW- thymus, trachea, esophagus, lungs, and heart), and perirenal fat were recorded for each carcass and their ratio to the CCW and reference carcass weight (RCW) calculated. Longissimus muscle was excised by removing the skin and connective tissue and stored at 4 °C until 48 h post-mortem for meat quality analysis, e.g., pH, color, cooking losses, and shear force.

#### 2.7.5. Meat Quality

The left longissimus muscle was used to measure pH, color, cooking losses, and shear force. Meat pH (pH48) and color were measured as previously referred. The cooking losses (%) were determined by individually placing meat samples inside polyethylene bags in a water bath at 80 °C for 1 h and cooled for 15 min under running tap water. The samples were then dried with filter paper and weighed. The cooking losses were expressed as the percentage of weight loss relative to the initial weight. After measuring the cooking losses, the samples were stored overnight in a refrigerator (4 °C). The meat samples used to determine the cooking loss were then cut into cuboid shape sub-samples (4) of 1 cm^2^ cross-section and 3–4 cm in length to determine the shear force, after room temperature equilibrium, using a Warner–Bratzler rectangular hole probe coupled to a TA.XT plus texturometer with a load cell of 30 kg/f (Stable Micro Systems, Godalming, UK). To perform these analyses, trigger force and blade velocity were set to 120 cm/min and 5 g respectively, and the sub-samples were placed with fibers perpendicular to the direction of the blade. Mean values for maximum shear force (N/cm^2^) over each sub-sample group were then obtained. 

#### 2.7.6. Fatty-Acid Profile

The right longissimus muscle of each animal was used to analyze the major fatty acids. The fatty acids were obtained using the protocol described by Bligh and Dyer [33] with modifications proposed by Barros [34]. Once the fatty acids were extracted, these compounds were transesterified according to the method described by Domínguez et al. [35], with some modifications. For sample preparation the method described by Munekata et al. [36] was used. The fatty acid methyl esters (FAMEs) were separated and quantified by gas chromatography (GC-Agilent 7890B, Agilent Technologies, Santa Clara, CA, USA) equipped with a PAL RTC-120 autosampler and a flame ionization detector (FID). The injection was carried out in split mode (1:50) with 1 L, the injector was kept at 260 °C, and the total flow was set to 64.2 mL/min. The separation was carried in an SP-2560 fused silica capillary column (100 m, 0.25 mm inner diameter (i.d.), 0.25-m film thickness; Supelco Inc., Bellefonte, PA, USA). The software MassHunter GC/MS Acquisition B.07.05.2479 (Agilent Technologies, Santa Clara, CA, USA) was used to control the equipment and perform the data analysis. Authenticated standards (FAME Mix, 37 components, docosapentaenoic acid, trans-vaccenic acid, cis-vaccenic acid, and CLA) were used to identify the FAMEs by comparing the retention times. The results were expressed as g/100 g of total identified fatty acids. After obtaining the fatty-acid data, the fractions of saturated (∑SFA), monounsaturated (∑MUFA), polyunsaturated (∑PUFA), and omega 3 and 6 (∑ω-3 and ∑ω-6) fatty-acid contents were determined, and the ratio ∑PUFA/∑SFA was calculated.

### 2.8. Statistical Analysis

Statistical analysis was carried out using the JMP program version 14 (SAS, 2018). Diet effect (C; 5U_GS_; 10U_GS_; 5T_GS_ and 10T_GS_) on the analyzed parameters was determined by analysis of variance (ANOVA). Orthogonal contrasts were performed to evaluate the effect of the incorporation of untreated or treated grape stalks vs. control diet, and the effect of the incorporation of untreated grape stalks vs. treated grape stalks (C vs. 5U_GS_ + 10U_GS_, C vs. 5T_GS_ + 10T_GS_, and U_GS_ vs. T_GS_). Differences were considered statistically significant at *p* < 0.05.

## 3. Results and Discussion

Experimental diets were similar in their chemical composition (Table 1) for CP (136.8 ± 3.2 g/kg, as fed), CF (41.8 ± 1.5 g/kg, as fed), gross energy (2252.4 ± 0.4 kcal/kg), and NDFom content (389.6 ± 8.8 g/kg, as fed). The inclusion of several ingredients (i.e., corn gluten feed, soybean, sunflower meal, rice bran, wheat straw, and soy husk) was not equivalent on all diets due to the different chemical composition of both untreated (U_GS_) and treated grape stalks (T_GS_). 

Overall, the experimental diets did not result in significant changes in animal performance, except daily weight gain (DWG), carcass weight (CW) and reference CW, lengths of the gastrointestinal tract, volatile fatty acids (VFA) profile, triglycerides and urea levels, and fatty acids profile. Data on growth performance and carcass traits are presented in Table 2. Higher daily weight gain (DWG) was observed on T_GS_ diets compared to the control diet (*p* = 0.034) and a trend (*p* = 0.069) for higher DWG was also observed compared to U_GS_. Rabbits fed on T_GS_ diets showed a lower feed conversion rate compared to rabbits fed on C and U_GS_ diets (*p* = 0.002 and *p* = 0.056, respectively). Carcass weight (CW) and reference CW were higher for the T_GS_ diets compared to the control diet (*p* = 0.038 and *p* = 0.030; respectively). Similar performance results were reported by several authors [37,38,39] in rabbits fed on diets containing fungi-treated substrates when compared to rabbits fed on diets including the non-treated substrates. El-Kady et al. [38] evaluated the replacement of hay for untreated corn stalks and those treated with *Trichoderma reesei* on rabbit’s diets and reported an increase of the DWG in an average of 28% for the treated cornstalks diet when compared to the control diet and 18% for diets containing untreated corn stalks. A similar trend was reported by Omer et al. [39] when studying the effect of the incorporation of rice straw treated with *P. ostreatus* and corn stalks with *T. reesei*, with rabbit’s fed on diets containing treated materials showing an increase in DWG of 11% compared to the control diet. According to Omer et al. [39], these positive effects on DWG could be attributed to secondary metabolites (such as exogenous enzymes, amino acids, or even vitamins) as a result of fungi mycelium growth on a substrate, and these can also influence the functionality of the gastrointestinal tract and feed efficiency [39]. The lower feed conversion ratio observed for T_GS_ diets also corroborates these findings (Table 2). However, data on CTTAD (Table 3) and the intestinal morphology of growing rabbits show that there were no differences between diets, indicating that other factors may have influenced the enhanced DWG and feed conversion rate. The morphology of villi and crypts are associated with gastrointestinal function, influencing the intestinal health status and its absorptive capacity. So, the gastrointestinal function and growth performance of rabbits are directly associated [40,41]. Results presented in Table 4 show that the length of caecum on rabbits fed with TGS diets is 5.4% longer compared to UGS diets (*p* = 0.015) and a trend (+4.7%) exists when compared to the control diet (*p* = 0.072). The same effect was observed for the small intestine measurements, with rabbits fed with TGS diets showing an increase of 5.6% (*p* = 0.021) compared to UGS diets (Table 4). Digestion and nutrient absorption mostly occur in the small intestine, which accounts for a large part of the total digestibility of dietary amino acid and starch, while the cecum is responsible for approximately half of the total digestive tract capacity [42]. The level and type of dietary fiber can play the most important role in controlling gastrointestinal tract development and digestive content and regulates feed intake and retention time in the caecum [43]. The caecal VFA concentration (Table 4) on rabbits fed with TGS diets showed a decrease in total VFA (−12.5%; *p* = 0.020), a decrease of acetic acid (−2.6%; *p* = 0.005), and an increase of butyric acid (−2.1%; *p* = 0.016) when compared with the incorporation of UGS, suggesting a different digestive efficiency between the experimental diets. VFA concentration is used as an indicator of microbial activity and significant dietary changes are required to modify VFAs due to their high variability [2]. For instance, the proportion of acetate increases, and that of butyrate generally decreases significantly when the fiber level increases [24]. Ribeiro et al. [21] showed the same trend of results for VFA profile when incorporating fungi-treated olive leaves in rabbit’s diets, suggesting that this increase could be the result of changes in the fiber chemical composition from fungi treatment. In this way, it seems that data obtained for DWG, feed conversion ratio, carcass weight (CW), and reference CW could be the result of the different digestive efficiency as well as the increase in the total available area for nutrient absorption. Data presented in Table 5 show that the experimental diets did not influence the major hematology and serum biochemistry parameters in rabbits’ blood.

Triglycerides were lower on animals fed with T_GS_ diets, showing a reduction of −11.9% when compared to U_GS_ diets (*p* = 0.005) and −19.3% compared to the control diet (*p* < 0.001). A slight decrease (*p* = 0.084) in the triglycerides of the rabbits fed with U_GS_ compared to the control diet was also detected (−8.5%). Studies indicate that winery wastes could be a valuable source of antioxidants and other active bio compounds, such as polyphenols and dietary fibers [44], that can reduce triglyceride levels in the bloodstream. Animal studies have been mainly focused on the utilization of grape seed extracts as a source of compounds that could attenuate hyperlipidemic effects. In rabbits, studies have pointed out that grape seed extracts can lower plasma triglyceride concentrations [45]. 

The lipid-lowering and anti-hyperlipidemic activity are normally attributed to the inhibition of the oxidation process of the low-density lipoproteins [46,47] due to the high concentrations of antioxidants [48]. As the phenolic substances concentration and the antioxidant activity of grape stems used in this study [49,50] are similar to the ones presented by grape seeds [51,52], it is feasible to assume that grape stems might also present the same anti-hyperlipidemic effects. Regarding urea, rabbits fed on U_GS_ and T_GS_ diets presented blood levels 13.5% and 19.3% lower than those fed on the control diet, respectively. Blood urea is the nitrogen content in urine from proteolysis, which occurs when energy production is insufficient to maintain the animals’ energy requirements [53]. The urea levels are also inversely related to dietary protein quality, i.e., the lower the serum urea levels, the better the quality of protein in the feedstuff [54,55]. In theory, the increased production of urea by the liver released into the blood for further excretion could be due to an unbalanced diet of any essential amino acids that would catabolize the remaining amino acids [54]. Although the experimental diets showed similar levels of crude protein (Table 1), their protein source is not the same due to the incorporation of T_GS_ and U_GS_, and this might have changed the amino acid profiles.

The fatty acids (FA) profile of *longissimus* muscle present in Table 6 shows that the rabbits fed with T_GS_ diets showed higher levels of linoleic acid (LA) when compared to the control diet (*p* = 0.016) and higher levels of γ-linolenic (GLA) when compared to the control diet (*p* = 0.026) and with U_GS_ diets (*p* = 0.019). It is well known that the FA levels of the monogastric animals’ meat are more susceptible to being manipulated through the diet since the FA are absorbed, unchanged, by the intestine and embedded tissues [56]. For example, linoleic acid, which plays an important role in functions such as cell physiology, immunity, and reproduction, is not synthesized de novo by animals and is thus required as a dietary source, given that concentrations present in the meat respond rapidly to changes in diet. In contrast, MUFAs and SFAs are synthesized and are less influenced by the diet than PUFAs [57]. Treatment with fungi on the grape stalks may have promoted an increase in LA and GLA since the deposition of these FA in meat is entirely derived from the diet. Fungi can produce a wide variety of lipids, with linoleic, oleic, and palmitic acids being the most reported fatty acids [58]. Some studies [59,60] already reported that *L. edodes* linoleic acid has the highest proportion in the lipid content (about 68%), followed by palmitic acid (16%) and oleic acid (5.5%). In this way, the increase in GLA could have been promoted by its presence on mycelium in the diet where fungi-treated grape stalks were incorporated. The T_GS_ diets also increased ∑ω-6, ∑PUFA, and ratio ∑PUFA/∑SFA, as a result of the increase of LA and GLA. PUFAs, in particular ω-3 and ω-6, are very important in human health due to their multiple biological roles, such as influencing the inflammatory processes, reducing the oxidative stress, neuroprotection, and cardiovascular protection [61]. The fungal treatment may have enhanced different lipid profiles on the diets, promoting an increase in both PUFA and the ratio of PUFA to SFA. No differences were observed on the analyzed parameters for meat quality (Table 7), such as pH, color, cooking losses, or even shear force. 

## 4. Conclusions

In summary, grape stalks (U_GS_ and T_GS_) could be effectively used as an alternative raw material in rabbits’ diets, representing a good strategy to optimize the use of winery by-products while maintaining animal performance. Fungi-treated grape stalks can provide some improvements, such as a decrease in plasma lipids in the bloodstream and an increase in fatty acids in rabbits’ meat, beneficial for human consumption. However, further studies with a greater number of animals are needed reinforce the obtained results and to understand specific metabolic routes due to the presence of fungal mycelium and their nutraceutical proprieties on rabbit nutrition.

## Figures and Tables

**Table 1:** Ingredients (g/kg, as fed) and chemical composition (g/Kg, as fed) of the five experimental diets.

	Experimental Diets				
	C	5U_GS_	10U_GS_	5T_GS_	10T_GS_
Ingredients (g/kg, as fed)					
Grape Stalk untreated	0	50	100	0	0
Grape Stalk treated	0	0	0	50	100
Alfalfa hay	180	180	180	180	180
Sunflower meal	200	185	115	180	125
Wheat	100	110	100	100	110
Beet pulp	80	80	100	100	80
Rice bran	50	50	10	50	10
Wheat bran	150	150	150	150	150
Wheat straw	70	15	15	15	10
Corn bran	20	20	25	40	25
Corn gluten feed	0	25	50	0	55
Palm kernel	60	60	55	60	55
Soy husk	30	15	0	15	0
Soybean	0	0	40	0	40
Sugarcane molasses	20	20	20	20	20
Minerals, vitamins and additives ^1^	40	40	40	40	40
Chemical composition (g/kg DM)					
Organic matter	899	908	908	909	900
Ash	101	92	92	91	100
NDFom	406	389	385	389	379
Total dietary fiber	455	462	465	474	472
Insoluble fiber	405	418	421	423	413
Soluble fiber	50	44	44	51	59
Crude protein	138	134	142	132	138
Ether extract	41	40	41	44	43
Gross energy (kcal/kg, as fed)	2252	2253	2253	2252	2252

C: control diet without grape stalks incorporation; 10U_GS_, 5U_GS_: diets with the incorporation of 100 and 50 g/kg of untreated grape stalks, respectively; 10T_GS_, 5T_GS_: diets with the incorporation of 100 and 50 g/kg of treated grape stalks with *L. edodes*, respectively; SEM: standard error of the mean; NDFom: neutral detergent fiber ash-free; ^1^: vitamin A, 10,000 IU; vitamin D3, 1080 U; vitamin E, 36 mg; vitamin K, 1 mg; vitamin B1, 2 mg; vitamin B2, 6 mg; vitamin B6, 2 mg; vitamin B12, 10 mg; niacinamide, 50 mg; Ca- pantothenate, 20 mg; folic acid, 5 mg; Fe, 78 mg; Cu, 14 mg; Co, 0.5 mg; Mn, 20 mg; Zn, 60 mg; Se, 0.05 mg; I, 1.1 mg; choline chloride, 260 mg. In addition, Calcium carbonate, Luctarom 1408-Z, L-Threonine, Toxmystat, Luctanox, Sepiolite, NL-510-R, salt, Biolys 70, Sodium bicarbonate and Bio-Mos.

**Table 2:** Effect of the experimental diets on growth performance and carcass traits (*n* = 10).

	Experimental Diets						*p*-Value		
	C	5U_GS_	10U_GS_	5T_GS_	10T_GS_	SEM	C vs. 5U_GS_ + 10U_GS_	C vs. 5T_GS_ + 10T_GS_	U_GS_ vs. T_GS_
Live weight at 35 d (g)	1094.4	1097.5	1094.9	1092.5	1077.0	18.4	0.936	0.671	0.538
Live weight at 66 d (g)	2554.7	2590.2	2578.5	2662.2	2612.0	40.3	0.551	0.102	0.197
Daily weight gain (g/d)	47.1	48.1	47.9	50.6	49.5	1.1	0.513	0.034	0.069
Daily feed intake (g/d)	152.0	148.8	150.3	151.0	149.5	3.0	0.506	0.642	0.805
Feed conversion rate	3.24	3.10	3.14	2.98	3.02	0.06	0.112	0.002	0.056
Carcass Weight (CCW), (g)	1394.8	1440.4	1426.9	1474.4	1424.4	20.9	0.136	0.038	0.455
Reference CCW (RCW; g)	1128.9	1176.6	1166.9	1203.5	1157.5	18.7	0.100	0.030	0.645
Carcass yield (%HCW)	54.7	55.6	55.4	55.4	54.6	0.54	0.204	0.617	0.342
RCW yield (%CCW)	80.9	81.7	81.8	81.6	81.2	0.38	0.100	0.304	0.439
Carcass color									
L*	44.4	44.5	43.9	41.8	44.3	1.17	0.903	0.370	0.342
a*	10.5	11.9	11.6	13.1	11.8	1.50	0.507	0.291	0.625
b*	12.7	14.6	13.3	13.7	13.2	0.76	0.191	0.426	0.524
pH_24_	5.85	5.86	5.84	5.96	5.83	0.06	0.994	0.550	0.459
Drip loss (%)	4.36	3.80	3.71	3.68	3.81	0.23	0.074	0.070	0.973
As % of CCW:									
Head	8.76	8.35	8.39	8.65	8.76	0.17	0.100	0.793	0.100
LHW	2.30	2.21	2.09	2.30	2.13	0.09	0.156	0.402	0.469
Liver	6.61	6.36	6.46	6.17	6.58	0.33	0.611	0.553	0.917
Kidneys	1.39	1.41	1.27	1.26	1.30	0.06	0.517	0.172	0.373
Perirenal fat	1.74	1.77	2.11	1.75	1.91	0.13	0.209	0.558	0.407
Scapular fat	0.81	0.72	0.84	0.80	0.74	0.04	0.590	0.431	0.780
Dissectible fat ^1^	3.18	3.28	3.60	3.34	3.30	0.17	0.231	0.518	0.496
As % of RCW:									
Fore part	38.9	40.0	38.0	38.5	39.2	0.82	0.901	0.989	0.866
Intermediate part	27.3	26.1	26.7	26.2	26.9	0.69	0.299	0.369	0.861
Hind part	40.5	39.8	39.9	39.5	39.8	0.45	0.280	0.156	0.670
*Longissimus muscle*	3.83	3.66	3.77	3.54	3.81	0.08	0.241	0.122	0.638
Pelvic fat	3.80	3.76	3.98	3.44	3.96	0.27	0.827	0.757	0.520

C: control diet without grape stalks incorporation; 10U_GS_, 5U_GS_: diets with the incorporation of 100 and 50 g/kg of untreated grape stalks, respectively; 10T_GS_, 5T_GS_: diets with the incorporation of 100 and 50 g/kg of treated grape stalks with *L. edodes*, respectively; SEM: standard error of the mean; L*: Lightness; a*: Redness; b*. Yellowness; LHW: Thymus, trachea, oesophagus, lungs, and heart; ^1^: Scapular, inguinal and perirenal fat. Differences were considered statistically significant at *p* ≤ 0.05.

**Table 3:** Effect of the experimental diets on coefficients of total tract apparent digestibility (CTTAD, *n* = 6) and intestinal morphology of growing rabbits (*n* = 10). Effect of the experimental diets on growth performance and carcass traits (*n* = 10).

	Experimental Diets						*p*-Value		
	C	5U_GS_	10U_GS_	5T_GS_	10T_GS_	SEM	C vs. 5U_GS_ + 10U_GS_	C vs. 5T_GS_ + 10T_GS_	U_GS_ vs. T_GS_
CTTAD (*n* = 6)									
Dry matter	0.62	0.59	0.59	0.60	0.59	0.01	0.078	0.120	0.561
Organic matter	0.62	0.59	0.60	0.60	0.59	0.012	0.102	0.180	0.731
NDFom	0.32	0.32	0.31	0.32	0.32	0.013	0.409	0.710	0.597
Crude protein	0.62	0.62	0.60	0.61	0.59	0.017	0.572	0.348	0.648
Crude Fat	0.86	0.85	0.85	0.86	0.87	0.010	0.308	0.719	0.111
Intestinal morphology (*n* = 10)									
Duodenum (µm)									
Villus height	1086.6	1137.0	1105.2	1110.0	1097.0	65.6	0.668	0.834	0.788
Villus tip width	117.0	125.4	117.6	119.4	117.3	4.44	0.417	0.806	0.487
Crypt depth	332.5	329.3	353.1	309.2	337.3	30.4	0.816	0.804	0.557
VH/CD	3.52	3.82	3.25	3.70	3.53	0.37	0.960	0.819	0.826
Muscle layer	215.3	191.0	187.1	195.9	174.7	14.6	0.148	0.100	0.800
Jejune (µm)									
Villus height	858.6	915.0	824.7	825.1	949.2	42.4	0.829	0.585	0.685
Villus tip width	118.9	125.6	118.4	120.3	126.5	5.42	0.639	0.500	0.800
Crypt depth	195.6	219.8	214.7	191.7	198.1	12.6	0.167	0.963	0.100
VH/CD	4.50	4.26	3.98	4.38	4.85	0.26	0.248	0.711	0.110
Muscle layer	196.6	163.8	177.9	186.1	163.0	11.3	0.069	0.117	0.746
Ileum (µm)									
Villus height	685.6	634.2	571.7	597.1	591.9	43.3	0.127	0.093	0.847
Villus tip width	119.3	118.8	111.0	119.6	126.2	5.50	0.518	0.595	0.153
Crypt depth	197.5	221.8	164.1	208.5	172.5	27.0	0.891	0.833	0.928
VH/CD	3.68	3.14	3.51	3.22	3.58	0.27	0.284	0.396	0.781
Muscle layer	238.9	225.2	283.1	218.9	248.7	18.0	0.492	0.818	0.264

C: control diet without grape stalks incorporation; 10U_GS_, 5U_GS_: diets with the incorporation of 100 and 50 g/kg of untreated grape stalks, respectively; 10T_GS_, 5T_GS_: diets with the incorporation of 100 and 50 g/kg of treated grape stalks with *L. edodes*, respectively; SEM: standard error of the mean; NDFom: neutral detergent fiber ash free; VH: Villus height; CD: Crypt depth.

**Table 4:** Effect of the experimental diets on pH of caecal content and stomach, gastrointestinal tract physiology, and total concentration and profile of volatile fatty acids (*n* = 10).

	Experimental Diets						*p*-Value		
	C	5U_GS_	10U_GS_	5T_GS_	10T_GS_	SEM	C vs. 5U_GS_ + 10U_GS_	C vs. 5T_GS_ + 10T_GS_	U_GS_ vs. T_GS_
pH Caecal content	5.93	5.79	5.97	5.74	5.91	0.06	0.443	0.119	0.323
pH Stomach	1.86	1.66	1.68	1.49	1.72	0.12	0.220	0.107	0.624
As % of HCW:									
Full caecum	10.6	10.1	10.5	10.9	10.4	0.55	0.734	0.947	0.620
Empty caecum	2.71	2.57	2.71	2.53	2.55	0.12	0.646	0.246	0.387
Stomach	7.37	8.57	8.41	7.91	9.26	0.52	0.086	0.063	0.853
Small Intestine	8.46	8.35	8.13	8.71	8.17	0.38	0.639	0.967	0.598
Colon	3.06	2.74	2.91	2.91	3.18	0.21	0.360	0.961	0.288
Length (cm):									
Appendix Ceacal	12.6	12.9	12.8	13.2	12.4	0.38	0.583	0.583	1.000
Caecum	50.1	49.6	50.0	53.2	51.8	1.07	0.665	0.072	0.015
Small Intestine	373.6	363.9	367.0	389.0	382.6	8.48	0.436	0.246	0.021
Colon	37.8	38.0	39.3	40.8	37.9	1.18	0.562	0.292	0.559
Total VFA (mmol/100 mL)	9.93	10.5	10.2	8.75	9.36	0.53	0.537	0.183	0.020
VFA profile (% of total VFA)									
Acetic acid	76.2	76.1	78.7	74.0	75.6	0.86	0.268	0.194	0.005
Propionic acid	5.30	4.78	5.09	5.14	5.39	0.28	0.279	0.918	0.231
Butyric acid	18.0	18.6	15.9	20.3	18.4	0.80	0.447	0.211	0.016
Valeric acid	0.47	0.52	0.38	0.49	0.58	0.09	0.809	0.586	0.337

C: control diet without grape stalks incorporation; 10U_GS_, 5U_GS_: diets with the incorporation of 100 and 50 g/kg of untreated grape stalks, respectively; 10T_GS_, 5T_GS_: diets with the incorporation of 100 and 50 g/kg of treated grape stalks with *L. edodes*, respectively; SEM: standard error of the mean; HCW: Hot Carcass Weight; VFA: volatile fatty acids. Differences were considered statistically significant at *p* ≤ 0.05.

**Table 5:** Effect of the experimental diets on blood hematology and serum biochemistry (*n* = 10).

	Experimental Diets						*p*-Value		
	C	5U_GS_	10U_GS_	5T_GS_	10T_GS_	SEM	C vs. 5U_GS_ + 10U_GS_	C vs. 5T_GS_ + 10T_GS_	U_GS_ vs. T_GS_
Haematology									
Erythrocyte (M/µL)	5.98	5.98	5.88	6.09	6.04	0.16	0.794	0.692	0.423
Haemoglobin (g/dL)	11.9	12.3	11.9	12.2	12.3	0.30	0.587	0.251	0.455
Haematocrit (%)	37.9	38.8	37.5	38.4	39.3	0.94	0.846	0.445	0.485
Lymphocytes (%)	60.5	63.8	58.7	64.4	62.6	2.96	0.850	0.414	0.440
Monocytes (%)	7.43	7.41	7.83	8.48	7.75	0.66	0.816	0.404	0.460
Eosinophils (%)	0.96	0.70	0.95	0.94	0.88	0.15	0.453	0.780	0.563
Basophils (%)	5.08	4.28	5.61	5.16	4.38	0.51	0.831	0.624	0.735
Reticulocyte count (%)	3.32	2.97	2.72	2.91	3.50	0.25	0.126	0.708	0.155
Serum biochemistry (mg/dL)									
Triglycerides	82.9	78.4	73.4	66.2	67.6	3.34	0.084	<0.001	0.005
Cholesterol	42.7	42.7	39.3	35.5	37.9	2.64	0.591	0.071	0.117
Urea	31.5	27.2	27.5	26.5	27.0	0.92	0.001	<0.001	0.510
Creatinine	0.75	0.75	0.59	0.75	0.75	0.05	0.227	0.652	0.122
AST	32.0	31.9	29.5	38.1	31.6	3.52	0.694	0.390	0.129
Alanine	31.7	39.8	33.6	33.8	34.5	4.04	0.318	0.623	0.531
Albumin	3.48	3.21	3.10	3.14	3.52	0.20	0.206	0.554	0.405
Total protein	4.54	4.75	4.47	4.55	4.56	0.09	0.507	0.886	0.523

C: control diet without grape stalks incorporation; 10U_GS_, 5U_GS_: diets with the incorporation of 100 and 50 g/kg of untreated grape stalks, respectively; 10T_GS_, 5T_GS_: diets with the incorporation of 100 and 50 g/kg of treated grape stalks with *L. edodes*, respectively; SEM: standard error of the mean; RDW: Red cell distribution width; AST: Aspartate aminotransferase. Differences were considered statistically significant at *p* ≤ 0.05.

**Table 6:** Effect of the experimental diets on fatty acids profile of longissimus muscle (g/100 g of fat; *n* = 10).

	Experimental Diets					*p*-Value			
	C	5U_GS_	10U_GS_	5T_GS_	10T_GS_	SEM	C vs. 5U_GS_ + 10U_GS_	C vs. 5T_GS_ + 10T_GS_	U_GS_ vs. T_GS_
SFA									
C4:0	0.10	0.11	0.12	0.12	0.11	0.01	0.484	0.357	0.785
C6:0	0.07	0.07	0.08	0.08	0.07	0	0.457	0.327	0.769
C8:0	0.05	0.05	0.06	0.06	0.06	0	0.413	0.342	0.869
C:10	0.11	0.12	0.12	0.12	0.11	0.01	0.447	0.431	0.973
C11:0	0.01	0.01	0.02	0.02	0.02	0	0.414	0.307	0.799
C12:0	0.63	0.58	0.58	0.64	0.6	0.03	0.21	0.756	0.247
C13:0	0.02	0.02	0.02	0.02	0.02	0	0.715	0.276	0.372
C14:0	2.11	2.02	2.08	2.14	2.10	0.1	0.612	0.932	0.469
C15:0	0.31	0.32	0.32	0.32	0.33	0.01	0.629	0.250	0.409
C16:0	18.23	18.2	18.97	18.43	18.16	0.58	0.615	0.927	0.614
C17:0	0.31	0.32	0.31	0.31	0.32	0.01	0.596	0.445	0.774
C18:0	3.69	3.76	3.75	3.74	3.64	0.11	0.626	0.988	0.563
C20:0	0.09	0.09	0.08	0.09	0.09	0	0.914	0.406	0.376
C21:0	0.02	0.02	0.02	0.02	0.02	0	0.962	0.233	0.162
C22:0	0.05	0.05	0.05	0.05	0.05	0	0.448	0.737	0.183
C23:0	0.08	0.08	0.08	0.09	0.08	0	0.665	0.796	0.831
C24:0	0.04	0.03	0.04	0.04	0.04	0	0.292	0.632	0.479
∑SFA	25.92	25.84	26.70	26.30	25.79	0.79	0.723	0.898	0.782
MUFA									
C14:1n–5	0.15	0.18	0.17	0.14	0.17	0.02	0.430	0.856	0.456
C15:1n–5	0.93	0.88	0.95	0.93	0.88	0.06	0.848	0.719	0.836
C16:1n–7	1.82	1.99	2.14	1.73	1.97	0.20	0.318	0.909	0.279
C18:1n–9t	0.12	0.13	0.13	0.13	0.13	0.01	0.546	0.342	0.670
C18:1n–11t	0.20	0.21	0.23	0.23	0.22	0.01	0.228	0.076	0.470
C18:1n–9	16.18	16.56	15.81	16.98	15.98	0.55	0.989	0.656	0.598
C18:1n–7	0.76	0.77	0.79	0.79	0.76	0.02	0.517	0.704	0.741
C20:1n–9	0.17	0.17	0.16	0.18	0.16	0.01	0.505	0.962	0.448
C22:1n–9	0.03	0.03	0.03	0.03	0.03	0	0.488	0.761	0.633
∑MUFA	20.37	20.93	20.4	21.13	20.31	0.71	0.738	0.691	0.937
ω-3 FA									
C18:3n–3	0.83	0.85	0.87	0.82	0.83	0.03	0.400	0.959	0.334
C20:3n–3	0.04	0.04	0.04	0.04	0.04	0	0.571	0.822	0.676
C20:5n–3	0.07	0.06	0.07	0.06	0.06	0	0.578	0.251	0.464
C22:5n–3	0.36	0.35	0.37	0.35	0.35	0.02	0.808	0.672	0.825
C22:6n–3	0.08	0.08	0.08	0.09	0.08	0.01	0.814	0.739	0.903
∑ω-3	1.38	1.39	1.41	1.36	1.37	0.03	0.548	0.792	0.291
ω-6 FA									
C18:2n–6	17.76	18.93	18.54	19.58	19.44	0.57	0.171	0.016	0.182
C18:3n–6	0.08	0.08	0.08	0.09	0.09	0	0.757	0.026	0.019
C20:2n–6	0.29	0.29	0.29	0.32	0.29	0.01	0.912	0.368	0.218
C20:3n–6	0.27	0.27	0.27	0.29	0.27	0.02	0.706	0.400	0.568
C20:4n–6	2.61	2.66	2.62	2.85	2.65	0.17	0.896	0.523	0.534
∑ω-6	21.00	22.23	21.81	23.13	22.74	0.63	0.200	0.017	0.157
Other PUFA									
C18:2n–9c,11t	0.16	0.17	0.19	0.18	0.17	0	0.285	0.199	0.786
∑PUFA	22.55	23.78	23.41	24.68	24.28	0.66	0.203	0.022	0.189
∑PUFA/∑SFA	0.87	0.92	0.88	0.94	0.94	0.02	0.292	0.014	0.072
∑FA	68.84	70.55	70.51	72.11	70.38	1.93	0.479	0.315	0.712

C: control diet without grape stalks incorporation; 10U_GS_, 5U_GS_: diets with the incorporation of 100 and 50 g/kg of untreated grape stalks, respectively; 10T_GS_, 5T_GS_: diets with the incorporation of 100 and 50 g/kg of treated grape stalks with *L. edodes*, respectively; SEM: standard error of the mean; SFA: saturated fatty acids; MUFA: monounsaturated fatty acids; PUFA: polyunsaturated fatty acids. Differences were considered statistically significant at *p* ≤ 0.05.

**Table 7:** Effect of the experimental diets on parameters of meat quality of longissimus muscle (*n* = 10).

	Experimental Diets							*p*-Value	
	C	5U_GS_	10U_GS_	5T_GS_	10T_GS_	SEM	C vs. 5U_GS_ + 10U_GS_	C vs. 5T_GS_ + 10T_GS_	U_GS_ vs. T_GS_
pH_48_	5.60	5.61	5.62	5.63	5.62	0.02	0.532	0.274	0.561
Meat color									
L*	59.6	59.5	59.1	60.2	59.3	0.73	0.726	0.868	0.527
a*	0.01	0.23	0.29	0.18	0.19	0.25	0.413	0.566	0.763
b*	9.78	9.61	9.54	10.2	9.86	0.23	0.475	0.394	0.059
Cooking losses (%)	30.3	30.0	30.7	29.5	30.9	0.79	0.981	0.907	0.863
Shear force	25.8	21.8	24.6	25.5	24.3	1.27	0.087	0.561	0.161

C: control diet without grape stalks incorporation; 10UGS, 5UGS: diets with the incorporation of 100 and 50 g/kg of untreated grape stalks, respectively; 10TGS, 5TGS: diets with the incorporation of 100 and 50 g/kg of treated grape stalks with *L. edodes*, respectively; SEM: standard error of the mean; pH48: pH measured 48 h after slaughter; L*: Lightness; a*: Redness; b*. Yellowness.

## Data Availability

The data are contained within the article.
